# The rapid endocytic uptake of fetuin‐A by adherent tumor cells is mediated by Toll‐like receptor 4 (TLR4)

**DOI:** 10.1002/2211-5463.13008

**Published:** 2020-11-03

**Authors:** Portia L. Thomas, Gladys Nangami, Tanu Rana, Adam Evans, Stephen D. Williams, Dylan Crowell, Anil Shanker, Amos M. Sakwe, Josiah Ochieng

**Affiliations:** ^1^ Department of Microbiology, Immunology and Physiology Meharry Medical College Nashville TN USA; ^2^ School of Graduate Studies and Research Meharry Medical College Nashville TN USA; ^3^ Department of Biochemistry, Cancer Biology, Pharmacology and Neuroscience Meharry Medical College Nashville TN USA

**Keywords:** adhesion, endocytosis, fetuin‐A, LPS, receptor, TLR4

## Abstract

Fetuin‐A is a serum glycoprotein synthesized and secreted into blood by the liver and whose main physiological function is the inhibition of ectopic calcification. However, a number of studies have demonstrated that it is a multifunctional protein. For example, endocytic uptake of fetuin‐A by tumor cells resulting in rapid cellular adhesion and spreading has been reported. The precise uptake mechanism, however, has been elusive. The present studies were done to determine whether Toll‐like receptor‐4 (TLR4), which has been previously shown to be a receptor for fetuin‐A and is commonly expressed in immune cells, could take part in the rapid uptake (< 3 min) of fetuin‐A by tumor cells. Rapid uptake of fetuin‐A was inhibited by the specific TLR4 inhibitor CLI‐095 and also attenuated in TLR4 knockdown prostate tumor cells. Inhibition of TLR4 by CLI‐095 also attenuated the rapid adhesion of tumor cells as well as invasion through a bed of Matrigel. The data suggest mechanisms by which TLR4 modulates the adhesion and growth of tumor cells.

## Graphical abstract

The study demonstrates that TLR4 mediates the rapid uptake of fetuin‐A by tumor cells to promote rapid adhesion, spreading, growth, motility, and invasion in serum‐free medium. The specific TLR4 inhibitor, CLI‐095, inhibits rapid fetuin‐A uptake, and consequently, most tumor cells, particularly those that do not synthesize fetuin‐A, fail to adhere, spread, or grow in serum‐free medium.

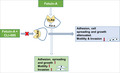

**Abbreviations**
ASF: asialofetuin
DMSO: dimethyl sulfoxide
FBS: fetal bovine serum
GFP: green fluorescence protein
HBS: Hanks buffered saline
HBSS: Hanks buffered salt solution
IMDM: Iscove’s modified Dulbecco’s medium
LPS: lipopolysaccharide
NEARS: Nikon Elements Advanced Research Software
NSC: nonsilencing control
PBS: phosphate‐buffered saline
SFM: serum‐free medium
TLR4: Toll‐like receptor 4

Fetuin‐A is a serum glycoprotein synthesized and secreted into blood by the liver and whose main physiological function is the inhibition of ectopic calcification [1]. However, a number of recent studies including from our laboratory have indicated that it plays a pivotal role in tumor initiation and progression [2, 3, 4]. Other studies have also demonstrated that it as a major serum protein in the sequelae of type‐2 diabetes [5, 6, 7]. Taken together, fetuin‐A is now categorized as a multifunctional protein in normal and pathophysiology of humans [8]. Studies indicate that for fetuin‐A to perform some of its physiological functions in tumor cells, it must be internalized. Inside the cells, fetuin‐A has the propensity to interact with a number of intracellular proteins such as histones [9]. We have demonstrated that fetuin‐A participates in the biogenesis of exosomes that promote adhesion, cell spreading, motility, and invasion [10]. Interestingly, tumor cells that synthesize and secrete fetuin‐A can grow albeit less vigorously, in serum‐free medium [11].

We have reported using labeled fetuin‐A that tumor cells readily take up the protein within 3 min at 37 °C [12]. Others have also demonstrated rapid uptake of fetuin‐A by smooth muscle cells [13], even though the uptake mechanism is yet to be defined. Uptake of labeled fetuin‐A could not be inhibited by excess unlabeled fetuin‐A as would be expected for a canonical cell surface receptor [11, 13]. This led to the speculation that perhaps it is taken up by other process such as macro‐ or micropinocytosis [13]. A recent report demonstrated that fetuin‐A is one of a handful of ligands for TLR4 and that it interacts with the extracellular motifs of the receptor via its terminal β‐galactosides [14]. This report prompted us to ask the question of whether TLR4 could take part in the uptake mechanisms of fetuin‐A in tumor cells. Toll‐like receptor‐4 is a member of the pattern recognition proteins, whose main established extracellular ligand is lipopolysaccharide (LPS) [15]. In resting immune cells, TLR4 is located in Golgi and plasma membrane. Myeloid differentiation factor (MD2) is an accessory protein that is essential for LPS‐mediated activation of TLR4. LPS on the other hand is delivered to TLR4 by another accessory protein CD14 [16]. Apart from LPS and fetuin‐A, other ligands that interact with TLR4 have been identified, including hyaluronan [17] and high‐mobility group box1 (HMB1) [18]. Signaling via Toll‐like receptors (TLRs) orchestrates mainly inflammatory responses that usually accompany innate host defenses against foreign‐microorganisms [19]. Interestingly, it is only recently that the exact endocytic uptake and processing mechanisms for TLR4, upon activation was defined [20].

More recent studies implicate TLRs in the progression of a number of tumors [21, 22, 23], where the inflammatory responses following the activation of these receptors by their natural ligands such as LPS are thought to be the drivers of transformation and tumor growth. The present study does not refute this dogma, but the data suggest that TLR4 participates in other fundamental adhesion and growth mechanisms of tumor cells.

Using a specific inhibitor for TLR4, CLI‐095, we demonstrated that at ~ 10 µg·mL^−1^ (100 µm), it inhibits the uptake of labeled fetuin‐A. We also knocked down TLR4 in tumor cells that express the receptor and assessed the abilities of parental nonsilencing controls as well as knockdown subclones to take up labeled fetuin‐A. We hereby show the interaction of fetuin‐A with TLR4 on the surfaces of adhered tumor cells. More importantly, we demonstrate that knockdown of TLR4 as well as CLI‐095 was able to effectively inhibit the uptake of labeled fetuin‐A by tumor cells. Taken together, the data show that TLR4 expressed by a variety of tumor cells plays a role in the rapid uptake of fetuin‐A by these cells.

## Materials and methods

### Cells

MDA‐MB‐231, BT‐549, and HCC1806 breast cancer as well as PC3 (prostate) and LN229 (glioblastoma) cell lines were purchased from ATCC, Manassas, VA. The breast cancer cell lines were cultured in Dulbecco minimum essential medium supplemented with 10% FBS, while PC3 and LN229 were maintained IMDEM supplemented with 10% FBS, containing 1× antibiotic/antimycotic (Life Technologies, Grand Island, NY, USA). The cells were maintained in humidified CO_2_ incubator at 37 °C. The serum‐free medium (SFM) used in the studies contained 1% (w/v) bovine serum albumin.

### Materials

Lipopolysaccharide (LPS) was purchased from Thermo Fisher, Waltham, MA, and CLI‐095 from InvivoGen, San Diego, CA, USA. All the other reagents unless otherwise stated were purchased from Sigma‐Aldrich, St Louis, MO, USA.

### Western Blot analysis

The expression of TLR4 as well as actin controls was performed as previously described [9]. Briefly, whole cell extracts from PC3, MDA‐MB‐231, and HCC1806 were prepared in radio‐immunoprecipitation assay (RIPA) buffer (50 mm Tris/HCl, pH 7.4, 1% NP‐40, 0.1% sodium deoxycholate, 150 mm NaCl, 1 mm EDTA, and freshly added protease and phosphatase inhibitor cocktail). Thirty micrograms of protein from each of the cell lines was loaded to 4–12% SDS/PAGE. The protein bands were transferred to a polyvinylidene fluoride membranes. After blocking with 5% nonfat milk, the membranes were incubated with antibodies to either TLR4 or β‐actin (Santa Cruz, Dallas, TX, USA) overnight at 4 °C. The membranes were exposed to chemiluminescence (PerkinElmer, PerkinElmer, Waltham, MA, USA), after incubation with the corresponding secondary antibodies for signal detection.

### Fetuin‐A uptake studies

Fetuin‐A purified as described [12] was labeled with rhodamine isothiocyanate as previously described [24]. The labeled protein was separated from unreacted label by passing twice through a desalting column (HiTrap™, GE Healthcare, Chicago, IL, USA). Cells were grown in chambered glass slides until ~ 80% confluent. Labeled fetuin‐A (1 mg·mL^−1^) was then added to the chambers (40 µg per chamber) in the absence (vehicle controls) or presence of CLI‐095 (10 µg·mL^−1^) and incubated for exactly 2 min. The cells in the chambers were washed once with Hanks buffered salt solution (HBSS) and then fixed in 4% formalin. The plastic chambers were removed and the slides cover‐slipped, examined under a confocal microscope and images acquired digitally. Mean arbitrary units of fluorescence plus or minus SD were quantified using Nikon Elements Advanced Research Software (NEARS). The experiment was repeated 3× using a different cell line each time.

### Fetuin‐A mediated cellular adhesion and spreading

In order to underscore the significance of rapid fetuin‐A uptake in anchorage‐dependent growth of tumor cells, the breast carcinoma cell line BT‐549 was allowed to adhere to plastic in Hanks buffered salt solution (HBSS) in the presence of only 1 mm Ca^2+^ ions. Under these conditions, integrins are not involved in the adhesion process because they require Mg^2+^ or Mn^2+^ ions. The cells (2 × 10^4^ cells per well) were allowed to adhere in 96‐well culture plates in HBSS (control); HBSS + fetuin‐A and HBSS + fetuin‐A + CLI‐095 for 2 h at 37 °C. The nonadherent cells were carefully removed and the loosely adhered cells fixed in 4% formalin for 15 min. The cells were then stained with crystal violet as described [25] and images acquired.

### Modulation of cell surface expression of TLR4 by lipopolysaccharide (LPS) and fetuin‐A in adhered tumor cells

Given that fetuin‐A is one of the ligands for TLR4, we questioned its potential to modulate the expression of TLR4 on the surfaces of adhered tumor cells by flow‐cytometry. To analyze the expression of cell surface TLR4, prostate cancer PC3 cells (~ 70% confluent) were serum starved for 24 h. Lipopolysaccharide was then added to the cells (10 µg·mL^−1^) in SFM either in the absence (control) or presence of purified fetuin‐A (2 mg·mL^−1^). The cells were incubated at 37 °C in humidified CO_2_ incubator for 2 h. They were then detached with 2 mm EDTA in PBS containing 0.1% (w/v) sodium azide to slow down translocation of the cell surface receptors. The cells were pellet and resuspended as single cells in cold FACS buffer (PBS containing 1% BSA and 0.1% sodium azide). They were then transferred to Eppendorf tubes (1 × 10^6^ cells per tube) and incubated without (unlabeled controls) or with Cy5‐labeled APC anti‐human CD284 (TLR4; BioLegend, San Diego, CA, USA; 10 µL per tube) for 30 min at 4 °C with end on end rotation. The cells were washed 3× with cold FACS buffer and analyzed by BD FACScalibur and the gates for samples determined by single color controls. Unlabeled control cells were used. Data were analyzed on flowjo software (TreeStar, TreeStar, San Carlos, CA, USA).

### Modulation of cell surface expression of TLR4 in adhered tumor cells by CLI‐095, a specific TLR4 inhibitor

Here, we questioned whether CLI‐095, a specific TLR4 inhibitor could modulate the cell surface expression of TLR4 in adhered and well spread tumor cells. To determine the cell surface expression, we allowed breast tumor cells, MDA‐MB‐231 to adhere in chambered glass slides in complete medium (with 10% fetal bovine serum) in the absence (vehicle controls) or presence of CLI‐095 (10 µg·mL^−1^) dissolved in DMSO for at least 24 h. To determine cell surface TLR4, the cells were fixed in 4% formalin for 30 min at room temperature. After washing twice with PBS, the cells were incubated with Cy5‐labeled anti‐TLR4 for 30 min at 37 °C, washed once with PBS, the chambers removed and a drop of slow‐fade added and cover‐slipped. The slides were then observed under confocal microscopy (Nikon A1R, Nikon, Melville, NY, USA) and images taken (4 separate slides for controls and 4 for treated slides). Intracellular TLR4 was also determined by fixing the cells in cold methanol instead of 4% formalin. The experiment was repeated using PC3 cells and TLR4 expression analyzed by FACScalibur as described above. Briefly, cells growing in T 75 culture flasks (~ 70 confluence) were serum starved for 24 h and then incubated in serum‐free medium containing (10 µL: 10 mL‐DMSO:SFM) DMSO vehicle control or CLI‐095 (10 µg·mL^−1^) in DMSO for 2 h in humidified CO_2_ incubator at 37 °C. The cells were detached using 2 mm EDTA in PBS containing 0.1% sodium azide, washed once with FACS buffer, and incubated with Cy5‐labeled APC anti‐human‐CD284 (TLR4) in Eppendorf tubes (1 × 10^6^ cells per tube) in FACS buffer for 30 min at 4 °C. The cells were then washed 3× in cold FACS buffer and analyzed as above.

### Dynamic uptake and localization of fetuin‐A and TLR4 in intracellular compartments in tumor cells

#### Knockdown of TLR4 in tumor cells

Prostate cancer PC3 cells were cultured in 6‐well plates until cell number approached ~ 2 × 10^5^ cells per well in 2 mL of DMEM/F‐12 and then transfected with nonsilencing control or human TLR4 shRNA in Lenti‐GFP using DNAfectin™ Plus as per the manufacture’s protocol (Applied Biological Materials Inc., Richmond, BC, Canada). The cells were selected in complete medium containing 2.5 µg·mL^−1^ of puromycin for 4 weeks. Puromycin‐resistant and GFP‐positive cells were further isolated using fluorescence‐activated sorting and thereafter maintained in selection medium. Knockdown was validated by western blot assays. The PC3 nonsilencing control cells (PC3‐NSC) and TLR4 knockdown (PC3‐C3) cells were added to glass‐bottom dishes and incubated for 24 h in complete medium. The medium was replaced with SFM (1 mL per dish) and rhodamine isothiocyanate‐labeled fetuin‐A (100 µg per dish) added and uptake monitored for 60 s (Nikon A1R). Fetuin‐A uptake studies were also undertaken using live glioblastoma LN229 cells. The cells were grown in glass‐bottom dishes (Matsunami Glass, Bellingham, WA, USA) until ~ 80% confluent. The cells were serum starved (in 1 mL of serum‐free medium) for 24 h and cells in the dishes prepared for live cell imaging (Nikon A1R). The serum starvation medium was replaced with fresh serum‐free medium (SFM), and labeled fetuin‐A (100 µg per dish) was added in the absence (control) or presence of CLI‐095 (10 µg·mL^−1^) at 0 time point and images (rhodamine) acquired at 0‐, 30‐, 60‐, and 120‐s time points. The control dish contained equivalent volume of DMSO (vehicle control).

#### Localization of fetuin‐A TLR4 in the intracellular compartments of adhered and spread tumor cells

MDA‐MB‐231 breast carcinoma cells were allowed to adhere to chambered glass slides in complete medium for 24 h. The medium was replaced with serum‐free medium and rhodamine isothiocyanate‐labeled fetuin‐A added to the chambers (100 µg per chamber) and incubated for 3 min at 37 °C, washed once with cold PBS, and fixed in cold methanol for 5 min. The cells were washed once more with PBS and incubated with antibodies to TLR4 followed by fluorescein isothiocyanate‐labeled secondary antibodies. The chambers were removed, a drop of antifade with DAPI added to the slides, cover‐slipped, and examined under a confocal microscope and images acquired.

### Adhesion and invasion of LN 229 cells in the presence of either CLI‐095 or asialofetuin‐A (ASF)

Detached LN229 cells were incubated in serum‐free medium without (controls) or with either CLI‐095 (10 µg·mL^−1^) or ASF (1 mg·mL^−1^) for 30 min at 37 °C and then allowed attach to plastic for 24 h in serum‐free medium without (vehicle controls) or with CLI‐095 (10 µg·mL^−1^) in DMSO or ASF (1 mg·mL^−1^), after which the adhered cells were fixed in 4% formalin and stained with crystal violet. The same cells, 250 000 cells per chamber in SFM without (controls) or containing CLI‐095, or ASF were added to the upper chambers (Boyden chamber invasion assay) while the lower chambers contained purified fetuin‐A (2 mg·mL^−1^) in SFM. After 24 h, the invading cells migrated to the underside of Matrigel‐coated polycarbonate filters. After removing the cells remaining in the upper chambers with cotton swabs, the migrated cells were fixed in 4% formalin and stained with crystal violet as described [24].

### Statistics

Statistical calculations were performed using GraphPad PRISM 5 software (GraphPad, San Diego, CA, USA). Data were expressed as mean plus or minus standard deviation. *P* values < 0.05 were considered significant.

## Results

### Uptake of fetuin‐A by tumor cells is attenuated by the TLR4 inhibitor, CLI‐095

**Fig. 1:**
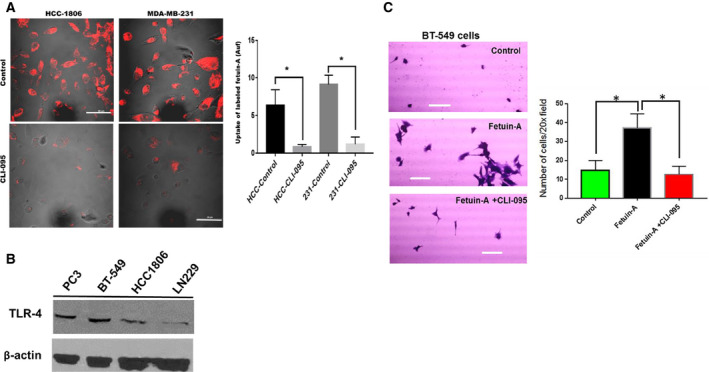
TLR4 mediates the uptake of fetuin‐A in adhered tumor cells. In (A), rhodamine isothiocyanate‐labeled fetuin‐A was added to the breast carcinoma cells in SFM attached to chambered glass slides (100 µg per chamber) in the absence (control) or presence of CLI‐095 (10 µg·mL^−1^) and incubated for 2 min at 37 °C. The medium was removed from the chambers, washed once with PBS, and cells fixed in 4% formalin. The chambers were removed and slides processed as described in Materials and methods. Fluorescence was quantified by NEARS (scale bar = 50 µm; *P < 0.05; *N* = 6; means compared by t‐test; error bars represent SD). In (B), the expression of TLR4 in the cell lines used in the study. In (C), BT‐549 breast carcinoma cells were allowed to adhere to plastic in Hanks buffered salt solution (HBSS) containing only [Ca^2+^] without (control) or with fetuin‐A (2 mg·mL^−1^) or fetuin‐A (2 mg·mL^−1^) + CLI‐095 (10 µg·mL^−1^) (*N* = 6; *P < 0.05; means compared by one way ANOVA, error bars represent SD). Scale bar = 50 µm.

We previously demonstrated that fetuin‐A is rapidly internalized by adherent tumor cells (~ 3 min) at 37 °C [12]. We now show that this rapid uptake is significantly attenuated by CLI‐095, the specific inhibitor of TLR4 in two breast carcinoma cell lines (Fig. 1A) both of which express TLR4 (Fig. 1B). The physiological consequence of rapid uptake of fetuin‐A by tumor cells is the biogenesis and secretion of exosomes that mediate rapid cell spreading and adhesion. This is better illustrated under conditions where integrins are minimally involved in the adhesion process. Hence, the cells are allowed to adhere loosely to plastic in the presence of Hanks buffered saline solutions containing only [Ca^2+^]. Under these conditions, control cells that do not synthesize and secrete fetuin‐A would not adhere or even spread on plastic. However, in the presence of added fetuin‐A, the cells spread and adhere loosely to plastic (Fig. 1C). This requires the rapid uptake of fetuin‐A, and therefore, the inhibition of the rapid uptake of fetuin‐A by CLI‐095 abrogates the adhesion and spreading of cells.

### Fetuin‐A downregulates TLR4 expression on the surfaces of adherent tumor cells

**Fig. 2:**
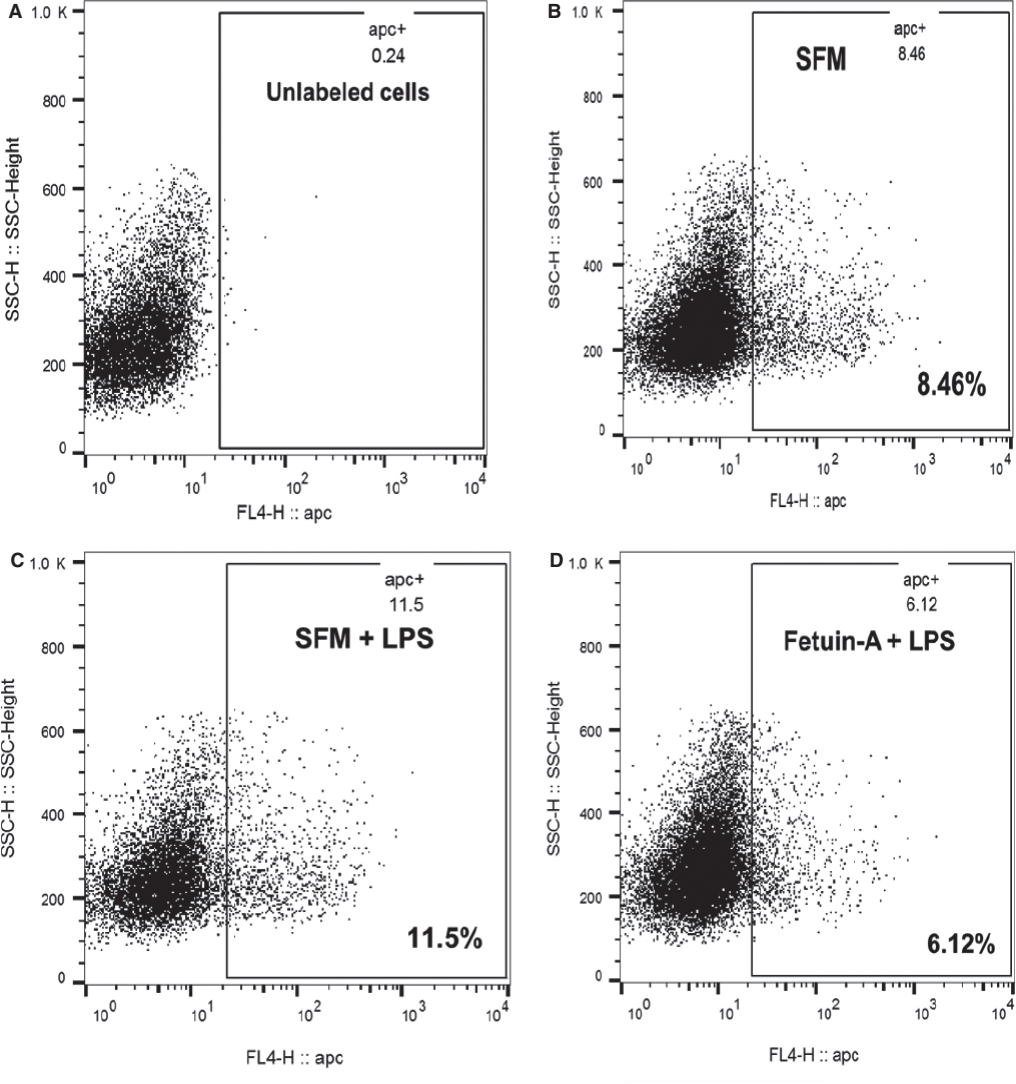
Fetuin‐A downregulates cell surface expression of TLR4 in adhered and spread cells. Prostate cancer PC3 cells were grown in 75‐CM^2^ culture flasks until ~ 70% confluent. Complete medium was replaced with serum‐free medium containing 1% BSA for 24 h. Serum‐free medium was replaced with fresh SFM without (B) or with lipopolysaccharide (LPS) (10 µg·mL^−1^) (C) or with LPS (10 µg·mL^−1^) and fetuin‐A (2 mg·mL^−1^) (D) and incubated at 37 °C in humidified CO_2_ incubator for 2 h. The cells were washed with PBS once and then detached with 2 mm EDTA in PBS containing 0.1% sodium azide. The cells were washed once with cold FACS buffer (PBS, 1% BSA, 0.1% NaN3), pelleted, and resuspended in 1 mL of cold FACS buffer in Eppendorf tubes (1 × 10^6^ cells per tube) and incubated without (A; unlabeled controls), or with CY5‐labeled anti‐human CD284 (TLR4) (10 µL per tube) for 30 min with end on end rotation at 4 °C. The cells were washed (3×) with cold FACS buffer and resuspended in the same buffer and analyzed by BD FACScalibur.

Given that fetuin‐A is one of the ligands of TLR4, we questioned whether fetuin‐A would influence the cell surface disposition of the receptor relative to its natural ligand, LPS in adherent cells. To do this, we employed Cy5‐labeled TLR4 monoclonal antibody. As shown in Fig. 2, serum‐free medium containing only 1% BSA supports the baseline surface expression of TLR4, and interaction with its natural ligand LPS, and appears to slightly upregulate its expression on the surfaces of adhered prostate cancer (PC3) cells. However, in the presence of purified fetuin‐A (2 mg·mL^−1^) and LPS, the surface expression of TLR4 in adherent cells PC3 cells was downregulated almost 2‐fold. The experiments were repeated 3× with similar data.

### TLR4 inhibitor, CLI‐095, traps the receptor on the cell surface

**Fig. 3:**
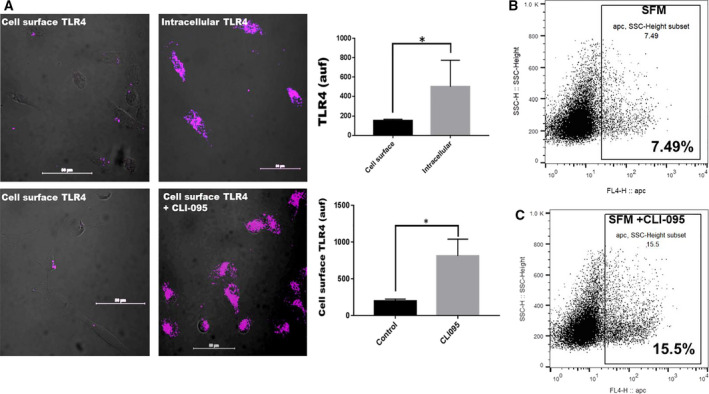
TLR4 is internalized in adhered and spread breast tumor cells. In (A), upper two panels. MDA‐MB‐231 breast tumor cells were allowed to adhere and spread (~50% confluence) in chambered glass slides in complete medium (10% fetal bovine serum) for 24 h. The slides were washed once with PBS and then cells fixed in either 4% formalin in PBS (cell surface TLR4) or cold methanol for 5 min (intracellular TLR4). The slides were then washed twice with PBS and the cells incubated with CY5‐labeled anti‐TLR4 for 30 min at 37 °C. The slides were washed with PBS, plastic chambers removed, and a drop of slow‐fade antifade added to the slides and cover‐slipped and images acquired (*N* = 4; *P < 0.05; means compared by *t*‐test; error bars represent SD). Fluorescence was quantified by NEARS. In the lower two panels, the breast tumor cells were also incubated in the chambered glass slides in complete medium for 24 h in the absence (control) or presence of CLI‐095 (10 µg·mL^−1^). The cells were then fixed in 4% formalin for 30 min at 37 °C to visualize cell surface TLR4, washed, and incubated with CY5‐labeled anti‐TLR4 and processed as above (*N* = 4; *P < 0.05; scale bar = 50 µm; means compared by *t*‐test; error bars represent SD). Prostate cancer cells (PC3) were also grown in culture until ~ 70% confluence in 75‐CM^2^ culture flasks. The complete medium was replaced with serum‐free medium and incubated for 24 h. The cells were then incubated with fresh SFM containing DMSO (vehicle control) (B) or CLI‐095 dissolved in DMSO for 2 h at 37 °C (C). The cells were detached with 2 mm EDTA in PBS containing 0.1% NaN_3_, washed in FACS buffer, and incubated with CY5‐labeled anti‐human CD284 for 30 min at 4 °C. The cells were washed 3× with cold FACS buffer and analyzed for TLR4 expression on BD FACScalibur.

Whereas the internalization of most cell surface receptors is a means of signal silencing, the internalization of TLR4 is a requirement for activation [20]. We therefore questioned whether the TLR4 inhibitor, CLI‐095, would interfere with its endocytic uptake (activation) and or redistribution and confine it to the cell surface in adherent cells. Adhered cells in the presence of purified fetuin‐A (2 mg·mL^−1^) or medium containing 10% FBS had negligible surface expression of TLR4. Most of the TLR4 were in intracellular compartments (Fig. 3A; upper panels). However, in the presence of fetuin‐A (2 mg·mL^−1^) or complete medium and CLI‐095 (10 µg·mL^−1^), cell surface expression of TLR4 was increased almost 10‐fold (*P < 0.05; Fig. 3A, lower panels), further suggesting that CLI‐095 attenuates the internalization of TLR4. The experiment was repeated with PC‐3 cells where the presence of CLI‐095 in SFM also resulted in a drastic increase in the cell surface expression of TLR4 (Fig. 3C) relative to SFM control (Fig. 3B).

### Time course of rapid uptake of fetuin‐A by tumor cells

**Fig. 4:**
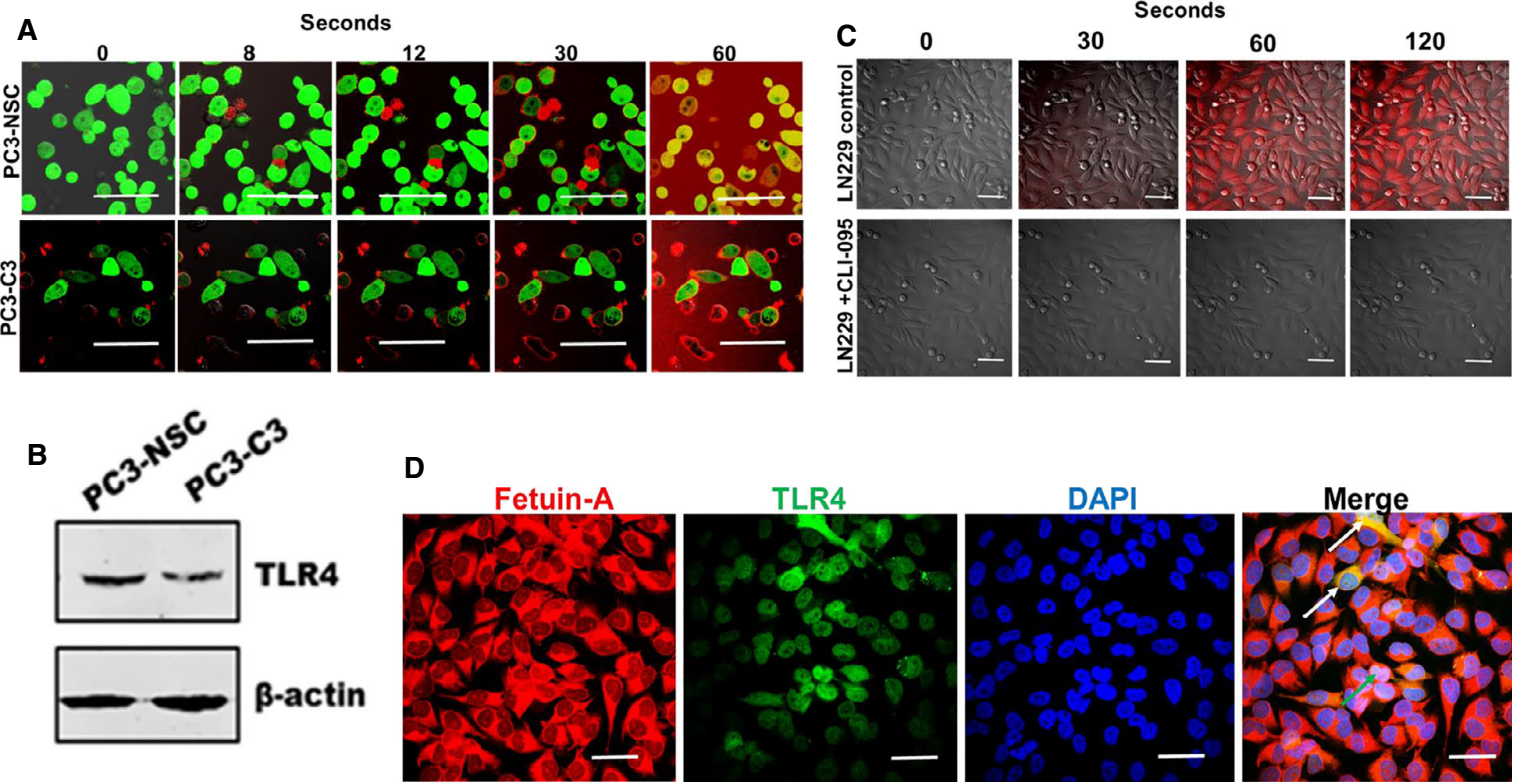
Dynamic uptake and localization of fetuin‐A and TLR4 in intracellular compartments in tumor cells. In (A), PC3 prostate cancer nonsilencing control (PC3‐NSC) and TLR4 knockdown (PC3‐C3) cells were added to glass‐bottom dishes and incubated for 24 h in complete medium. The medium was replaced with SFM (1 mL per dish) and rhodamine isothiocyanate‐labeled fetuin‐A (100 µg per dish) added and uptake monitored for 60 s (Nikon A1R). In (B), the expression of TLR4 in PC3‐NSC and PC3‐C3. In (C), the glioblastoma LN229 cells were added to glass‐bottom petri dishes in complete medium and incubated for 24 h at 37 °C (~ 80% confluence). The medium was replaced with SFM (1 mL per dish) containing either 100 µL per dish of DMSO (vehicle control) or CLI‐095 (10 µg·mL^−1^; 100 µL per dish). To control and experimental dishes, 100 µg per dish of rhodamine isothiocyanate‐labeled fetuin‐A was added at time 0 s and uptake by the live cells monitored using confocal microscope (Nikon A1R) for up to 120 s. In (D), rhodamine isothiocyanate‐labeled fetuin‐A was added to MDA‐MB‐231 breast carcinoma cells adhered to glass‐chambered slides, incubated for 2 min at 37 °C, washed once with cold PBS, and fixed in cold methanol for 5 min. The cells were washed once more with PBS and incubated with antibodies to TLR4 followed by fluorescein isothiocyanate‐labeled secondary antibodies (green). The chambers were removed and the slides processed as described in Materials and Methods. Scale bars = 100 µm.

We next wanted to determine the fetuin‐A uptake in real time both in the TLR4 knockdown PC3 cells and in the LN229 glioblastoma cells in the absence and presence of CLI‐095. The fetuin‐A uptake in nonsilencing control PC3 cells was quite rapid (within 60 s) but not in the TLR4 knockdown (PC3‐C3) where the labeled fetuin‐A was concentrated on the cell surface instead (Fig. 4A). The expression of TLR4 in PC3 and its knockdown subclone is depicted in Fig. 4B. The clones in which the TLR4 knockdown was > 80% did not survive beyond passage 1, underscoring the significance of TLR4 in the growth of tumor cells. The fetuin‐A uptake was similarly rapid in control LN229 glioblastoma cells (~ 60 s). However, in the presence of CLI‐095 as suspected, there was no uptake even after 120 s (Fig. 4C). Both fetuin‐A and TLR4 are localized mainly in intracellular compartments (perinuclear) in adhered and spread cells. They are clearly colocalized in some cells (yellow; white arrows) while in other cells they appear to be colocalized in the nucleus (white; green arrow; Fig. 4D). Interestingly, the appearance of both fetuin‐A and TLR4 in the nucleus has been reported [9, 25].

### TLR4 inhibitor, CLI‐095, attenuates both adhesion and invasion potentials of glioblastoma cell line, LN229

**Fig. 5:**
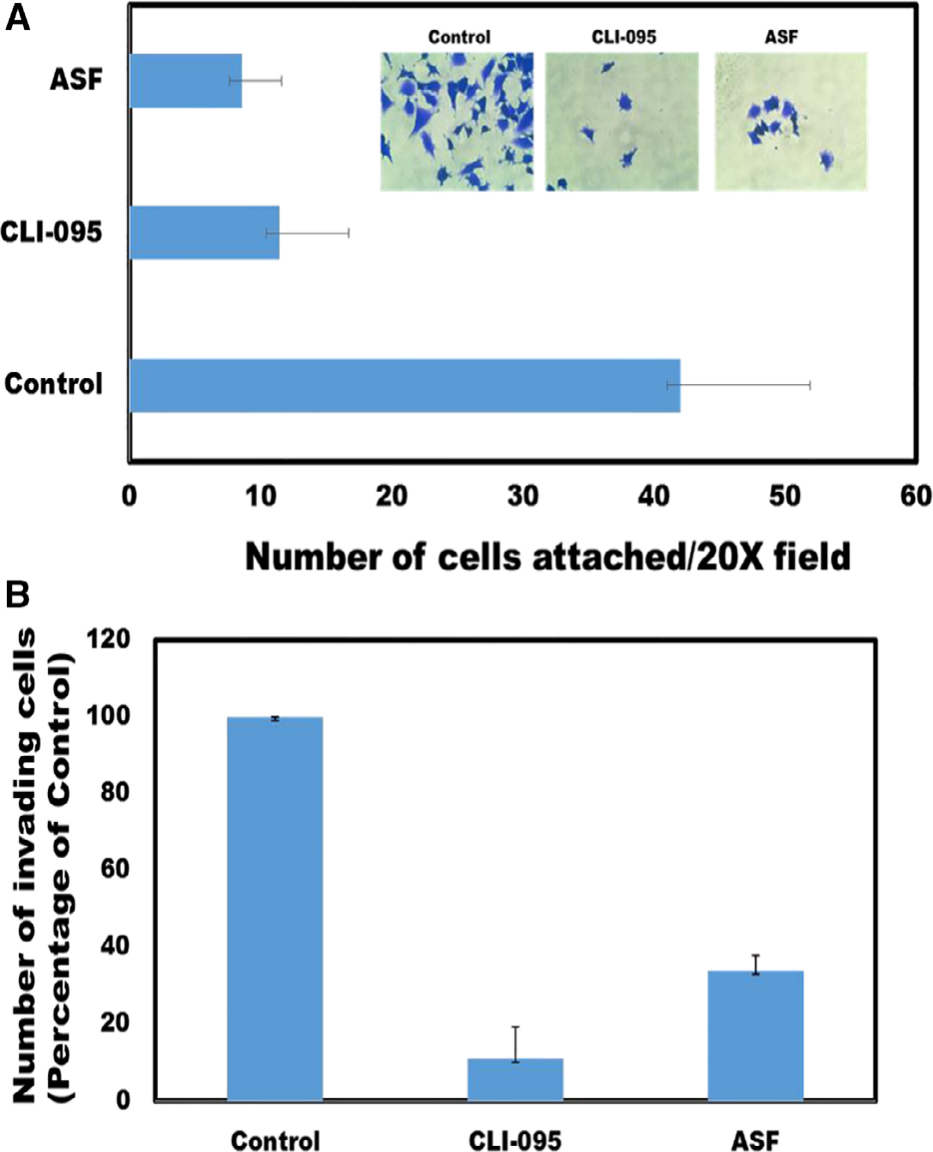
Incubation of detached LN229 cells with TLR4 inhibitor and ASF attenuates their attachment to plastic and invasion through Matrigel. In (A), detached LN229 cells were incubated in SFM without (control) or with CLI‐095 (10 µg·mL^−1^) or asialofetuin‐A (ASF) (1 mg·mL^−1^) for 30 min and then allowed to attach to plastic for 24 h under the same conditions. In (B), LN229 cells (250 000 cells per chamber) in SFM without (controls) or containing CLI‐095 (10 µg·mL^−1^) or ASF (1 mg·mL^−1^) were added to the upper chambers (Boyden invasion assay) and the lower chambers contained purified fetuin‐A (2 mg·mL^−1^) in SFM. After 24 h, the invading cells migrated to the underside of polycarbonate filters in 20× microscopic objective field were counted. The invading cells in the treatment groups were expressed as percentage of untreated control cells. Error bars represent SD.

Incubation of the detached cells with CLI‐095 or asialofetuin‐A (ASF) severely compromised their abilities to adhere and spread on plastic (Fig. 5A). LN229 synthesize and secrete fetuin‐A [11]. In the controls, the fetuin‐A can then be taken up via TLR4 to promote adhesion, while in CLI‐095‐treated cells, fetuin‐A uptake is inhibited and consequently adhesion is attenuated. Previous data suggested that asialofetuin‐A competed with fetuin‐A for binding to the putative receptor [11]. Given that ASF has terminal β‐galactosides that are not hindered by the sialic acid residues, it appears to interact and bind more efficiently to TLR4. We demonstrated that it also enters tumor cells rapidly and acts as a dominant‐negative inhibitor of fetuin‐A‐mediated functions in tumor cells [11]. The presence of either CLI‐095 or ASF in the upper chambers of the Boyden apparatus attenuates the capacity of the cells to interact with the Matrigel, thereby slowing and minimizing the process of invasion (Fig. 5B).

## Discussion

We previously demonstrated the rapid uptake of fetuin‐A by tumor cells which resulted in the biogenesis of exosomes that mediated adhesion, motility, and invasion [10]. We, however, were not able to elucidate the mechanism(s) by which fetuin‐A was rapidly internalized. We hereby provide experimental evidence that TLR4 participates in the rapid uptake of fetuin‐A by tumor cells resulting in rapid attachment and spreading of cells on substrata. The experiments were performed using multiple tumor cell lines to underscore the significance of fetuin‐A and TLR4 in the *in vitro* growth of these cells.

The abrogation of rapid endocytic uptake of fetuin‐A by the TLR4 inhibitor, CLI‐095 as well as knockdown of the receptor in tumor cells, demonstrates that TLR4 plays a central role in the physiological functions of fetuin‐A particularly in tumor cells. Of note was the fetuin‐A‐mediated downregulation of the surface expression of TLR4 in the adhered and spread tumor cells. Lipopolysaccharide (LPS), the natural ligand of TLR4 by itself, was not able to promote the translocation of the TLR4 from the cell surface to endosomal compartments as expected. Thus, the low surface expression of TLR4 on the surfaces of tumor cells growing in culture plates could be due to fetuin‐A which is present in most growth media supplemented with 10% fetal bovine serum. The data suggest that fetuin‐A and TLR4 comigrate into intracellular compartments. The rapid uptake of fetuin‐A by adherent tumor cells is necessary for their growth and survival. Reduced levels or absence of fetuin‐A in the growth media or tumor microenvironment results in slow and poor growth of tumor cells *in vitro* and *in vivo* [3, 4, 11]. Fetuin‐A mediates optimal cellular adhesion and spreading on various substrata not only *in vitro* but also *in vivo*, thereby promoting anchorage‐dependent cellular proliferation. The rapid uptake of fetuin‐A initiates the biogenesis and secretion of exosomes that promote cell spreading and rapid adhesion [10, 26].

The accumulation of TLR4 on the surfaces of adherent cells in the presence of its inhibitor, CLI‐095, provided further evidence that activated TLR4 drives the uptake of fetuin‐A into intracellular compartments. CLI‐095, which associates with the cytosolic domain of the receptor, inactivates it, and consequently, it is not endocytosed [20]. In immune cells, LPS first binds to the GPI‐anchored CD14 which then transfers LPS to cell surface MD‐2 which then dimerizes TLR4 to be trafficked into endosomal compartments followed by the activation process of TLR4 [20]. Because fetuin‐A also interacts with extracellular domains of TLR4 [14], it is tempting to speculate that in the absence or low levels of expression of MD‐2, fetuin‐A may promote the dimerization of TLR4 as a primer for endocytic uptake where the two molecules comigrate into endosomal compartments. The present studies made it clear that activation of TLR4 is essential for not only the trafficking of TLR4 into the intracellular compartments including the nucleus, but also for fetuin‐A which can also be trafficked into the nucleus [9]. The role of fetuin‐A in the growth of cells in culture either directly or indirectly has been debated ever since its discovery over 70 years ago [5, 27, 28, 29]. Assuming that the rapid uptake of fetuin‐A by cells is part of its growth‐promoting process, the present data suggest that TLR4 plays other functional roles in tumor cells that do not involve inflammatory responses. Indeed, a number of studies have implicated TLR4 to be involved in key growth and antiapoptotic signaling mechanisms in tumor cells [30, 31].

The potential role(s) of fetuin‐A in tumor progression is currently of significant interest. Recently, a system biological approach study indicated that fetuin‐A is a significant molecular determinant of metastasis [32]. Fetuin‐A has also been suggested to be a determinant of prostate cancer metastasis [2] and a key molecule in the etiology of male breast cancer [33]. In Boyden chamber assays for motility and invasion, medium containing 10% fetal bovine serum is generally added to the bottom chambers as chemoattractant. We previously reported that purified fetuin‐A at a concentration of ~ 2 mg·mL^−1^ can also be used as a chemoattractant in such assays [34]. Interestingly, the concentration of fetuin‐A in medium containing 10% fetal bovine serum is ~ 2 mg·mL^−1^.

In summary, we have provided evidence that TLR4 participates in the rapid uptake of fetuin‐A by tumor cells and that inhibition of TLR4 activation by a specific inhibitor of the receptor attenuates the fetuin‐A mediated physiological events in tumor cells such as rapid adhesion, cell spreading, invasion, and growth on the substrata.

## Conflict of interest

The authors declare no conflict of interest.

## Author contributions

PLT, GN, and JO conceived, designed, performed experiments, analyzed data, and wrote the manuscript; PLT and GN contributed equally to this work. TR, AE, SW, and DC performed experiments; AS and AMS provided new tools and reagents and supervised the study.

## Data Availability

Data will be available from the corresponding author upon request.
